# The Points of a Draught-horse

**Published:** 1902-01

**Authors:** 


### THE POINTS OF A DRAUGHT-HORSE.

HAVE the head of a fair size. Do not buy a horse with a pony head. A wide forehead is a good indication in a colt, the poll being not so wide, as the ears tend to droop, such horse often being stubborn in disposition. The eye should be prominent and the throat-latch clean, with a well-muscled neck. The shoulders should be somewhat sloping. Upright shoulders tend to make the gait stilted.

While good width in front is necessary, the legs should not be placed to the outside of the body. Such horses lack the straight-away gait and tend to roll. The knees should be broad and the tendons at the back of the legs well defined. The pasterns should be lengthy and set well back; the foot large, tough, with no bones.

At the heart girth the horse should be deep and full, with a short back and broad loin. The quarters should be long and level, with heavily muscled thighs. Especially important are the hocks, which should be clean, broad, and free from fleshiness, close together. No good draught-horse goes wide at his hocks. A colt going wide will tend to go wider with age, and consequently loses propelling power. The limb from heel to fetlock should be perpendicular—no curby hocks.

The skin should be clean and the hair fine of the model draught-horse, who must not paddle, turn the toes either in or out, and should show the sole of the foot at each step.—*Agricultural Journal*, October 24, 1901.

